# Gene Expression Profiles of Human Mesenchymal Stromal Cells Derived from Wharton’s Jelly and Amniotic Membrane before and after Osteo-Induction Using NanoString Platform

**DOI:** 10.3390/cimb44090291

**Published:** 2022-09-14

**Authors:** Vun Vun Hiew, Haselamirrah Mohd Akhir, Peik Lin Teoh

**Affiliations:** Biotechnology Research Institute, Universiti Malaysia Sabah, Jalan UMS, Kota Kinabalu 88400, Malaysia

**Keywords:** amniotic membrane, Wharton’s jelly, osteogenesis, signalling pathways, mesenchymal stem cells

## Abstract

The use of perinatal mesenchymal stem cells (MSCs) in bone tissue regeneration and engineering to substitute bone marrow MSCs has drawn great interest due to their high yield, ease of procurement, multilineage differentiation potential and lack of ethical concerns. Although amniotic membrane (AM) and Wharton’s jelly (WJ)-derived MSCs have been widely shown to possess osteogenic differentiation potential, the intrinsic properties determining their osteogenic capacity remain unclear. Here, we compared gene expression profiles of AM- and WJ-MSCs at basal and osteogenic conditions by using the NanoString Stem Cell Panel containing regulatory genes associated with stemness, self-renewal, Wnt, Notch and Hedgehog signalling pathways. At basal condition, WJ-MSCs displayed higher expression in most genes regardless of their functional roles in self-renewal, adhesion, or differentiation signalling pathways. After osteo-induction, elevated expression of self-renewal genes *ADAR* and *PAFAH1B1* was observed in AM-MSCs, while stemness genes *MME* and *ALDH1A1* were upregulated in WJ-MSC. Both MSCs showed differences in genes associated with ligands, receptors and ubiquitin ligases of the Notch pathway. In addition, further evidence was demonstrated in some signalling molecules including *CTBPs*, protein kinases, phosphatases, *RHOA*, *RAC1*. Downstream targets *HES1* and *JUN* especially showed higher expression in non-induced WJ-MSCs. Hedgehog genes initially expressed in both MSCs were downregulated in WJ-MSCs during osteogenesis. This study has provided insights into the intrinsic biological differences that may lead to their discrimination in therapeutic intervention.

## 1. Introduction

Osteoporosis is a bone disease characterized by low bone mass density and bone tissue degeneration which affects more than 200 million people worldwide. The treatment for osteoporosis involves the balancing of bone homeostasis that requires bone remodelling and osteogenesis that leads to new bone formation [1]. Mesenchymal stromal cells (MSCs) are an ideal source for cell replacement to treat bone diseases due to their distinct properties such as self-renewal and their ability to differentiate into multiple types of cells including bone, cartilage, and adipocyte [2,3].

Although BM-MSCs have been extensively studied for bone regeneration, MSCs from the placenta have become alternative sources because of their ease of accessibility without invasiveness, freedom from ethical concerns, and their MSC properties do not decline with age. The placenta is comprised of a variety of tissues including amniotic membrane (AM), Wharton’s jelly (WJ), chorionic membrane and decidua. AM- and WJ-MSCs were shown to have high proliferative and remarkable osteogenic differentiation potential [4,5]. Their applications have even grown from preclinical to clinical studies for a wide spectrum of medical treatments including bone regeneration and healing [6,7]. The osteogenic differentiation potential of AM- or WJ-MSCs either alone or applied in conjunction with a biomaterial have been well established [8,9].

Many studies have demonstrated that MSCs possess similar biological characteristics but have distinct paracrine, gene or protein expression profiles [10,11]. For example, WJ-MSCs were found to have inferior osteogenic commitment compared to BM-MSCs due to their low and late expression of osteogenic markers upon osteogenesis [12]. This was associated with the decreased expression of WISP1 involved in the Wnt pathway [13]. Nevertheless, the addition of BMP2 was capable of priming and promoting the osteogenic differentiation of WJ-MSCs [12]. This presents a hint that different sources of MSCs with eminent intrinsic expression profiles may affect the outcomes of clinical applications. It is important to find the most suitable source of MSCs and their appropriate osteogenic state to treat bone diseases.

In addition, MSCs give rise to osteoblasts to form bone, and this commitment is regulated by complex and interconnected signalling pathways such as Wnt, Notch and Hedgehog (Hh) that interconnect with each other to stimulate the targeted response [14]. Wnt signalling is known to promote osteogenesis in MSCs at the expense of adipogenic differentiation [15]. Notch signalling modulates cell survival, proliferation, differentiation, and fate determination in MSCs and has been shown to promote MSC osteogenesis via BMP/Smad signalling [16]. However, the activation of Notch in promoting osteogenic differentiation is dose- and context-dependent [17]. Hh signalling is a key regulator for bone development and is shown to promote osteoblast differentiation in human BM-MSCs by upregulating the expression of RANKL [18]. However, research is still lacking comparisons between the genes associated with these signalling pathways differentially expressed in AM- and WJ-MSCs during osteogenesis.

Although MSCs from AM and WJ possess osteogenic differentiation potential, the intrinsic gene expression determining their osteogenic capacity remains unclear. In this study, we performed comparisons of different signalling pathways and transcription factors involved in regulating the characteristics of undifferentiated and osteo-induced AM and WJ-MSCs by using the NanoString platform.

## 2. Methods

### 2.1. Samples

Human placentae were collected from KPJ hospital, Kota Kinabalu, Malaysia. Amniotic membrane and umbilical cord (UC) samples were obtained after caesarean delivery. The study was ethically approved by the Ethics and Research Committee of Universiti Malaysia Sabah with the approval code of JKEtika 1/16 (1). The informed consent for the placenta donation was taken from all neonate mothers.

### 2.2. MSCs Cultures

WJ-MSCs isolated from the UC matrix were obtained using enzymatic digestion or explant method. UC was first cut into small sections (4–5 cm in length) and rinsed with 1× Dulbecco’s phosphate buffered saline (DPBS) (Gibco-Invitrogen, Carlsbad, CA, USA). After the removal of umbilical veins and arteries, WJ tissues were peeled off carefully from the outlining membrane of the cord and chopped into small pieces. For the enzymatic method, they were digested with 0.1% collagenase type I (Worthington, MN, USA) at 37 °C for 2 h with constant agitation at 180 rpm. Cell suspension was centrifuged at 5000 rpm for 5 min at room temperature. The resulting cell pellet was resuspended using culture media and transferred into a 6-well plate for culturing. For the explant method, WJ was transferred onto the surface of Petri dishes after being cut into pieces about 3–4 mm each. The medium was DMEM/F12 (Gibco-Invitrogen, Carlsbad, CA, USA) supplemented with 10% foetal bovine serum (Gibco-Invitrogen, Carlsbad, CA, USA), 1% GlutaMAX (Gibco-Invitrogen, Carlsbad, CA, USA), 1× antibiotic-antimycotic (Gibco-Invitrogen, Carlsbad, CA, USA), and 1% ascorbic acid (Merck, Darmstadt, Germany). Cell culturing was performed at 37 °C in a humidified atmosphere with 5% CO_2_ and media was changed every 3–4 days. When the cells reached 80–90% confluency, they were detached using 0.25% trypsin-EDTA (Gibco-Invitrogen, Carlsbad, CA, USA). Cell pellets were harvested and seeded at a density of 1 × 10^5^ cells/cm^2^ in T25 flask for cell expansion. The enzymatic method was used to isolate AM-MSCs. The process and culturing condition were described in [19].

### 2.3. MSCs Differentiation

For differentiation assays, 3 × 10^4^ cells were seeded in 6-well plates. After cell attachment, the media was changed to osteogenic or adipogenic media. The StemPro Osteogenesis Differentiation Kits were purchased from Gibco-Invitrogen (Carlsbad, CA, USA). Media were replaced with fresh media every three days. Differentiation was carried out for ten days and 21 days for WJ-MSCs and AM-MSCs, respectively. The duration of osteogenic differentiation was determined using Alizarin Red solution (ScienCell Research Laboratories, Carlsbad, CA, USA). The cells were washed with 1× DPBS (Gibco-Invitrogen, Carlsbad, CA, USA) before being subjected to RNA extraction or osteogenic differentiation assay. For osteogenic differentiation assay, cells were fixed with 4% formaldehyde (Merck, Darmstadt, Germany) for 30 min at room temperature and then rinsed with 1× DPBS (Gibco-Invitrogen, Carlsbad, CA, USA). Formation of osteocytes was assessed using 2% Alizarin Red solution (ScienCell Research Laboratories, Carlsbad, CA, USA).

### 2.4. RNA Extraction

WJ- and AM-MSCs were seeded in 6-well plates with a density of 3 × 10^4^. After osteogenic differentiation, total RNA was extracted using a TransZol Up Plus RNA extraction kit (Transgen Biotech, Beijing, China) according to the manufacturer’s protocols. RNA concentration was measured using a Nanodrop 2000 spectrophotometer.

### 2.5. Sample Preparation for nCounter Stem Cell Panel

The mRNA transcript levels were detected using a Human nCounter^®^ Stem Cell Panel (NanoString Technologies, Seattle, WA, USA). RNA samples were prepared according to the manufacturer’s protocols and performed at University of Malaya, Malaysia. Briefly, 100 ng of total RNA from each sample was subjected to hybridization. Initially, a master mix was prepared by adding 70 µL of hybridization buffer to the Reporter Codeset. Next, 8 µL of the master mix and 5 µL from the sample were added to each hybridization tube. To complete the hybridization reactions, 2 µL of Capture Probeset were added to each tube. The reagents were mixed by inverting the tubes a few times before placing them into the 65 °C thermal cycler. The reactions were incubated for 16 h, then proceeded to the nCounter Prep Station. The detailed protocols can be obtained from https://nanostring.com/support/support-documentation/ (accessed on 8 August 2022).

### 2.6. Data Analysis

Samples with a count number of less than 100 were considered undetectable and excluded from the analysis. The number of qualified samples subjected to analysis were three and two for each WJ and AM group, respectively. Gene expression comparison was performed after normalization of the housekeeping genes *CLTC*, *GAPDH*, *GUSB*, *HPRT1* and *TUBB*. Fold change was obtained using NanoString nSolver Analysis software version 4.0. The filtering criteria for differential expressed genes was ±1.5-fold change. Significant differences between WJ- and AM-MSCs in the non-induction state were determined by the Student’s *t*-test. The *p* values of <0.05 and <0.005 were considered statistically significant.

## 3. Results

### 3.1. Differential Gene Expression of AM- and WJ-MSCs under Basal Condition

**Figure 1:**
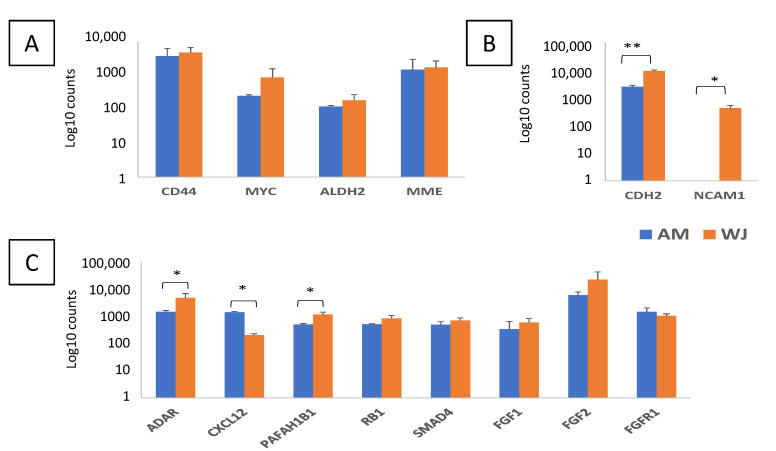
Differential expression of genes associated with self-renewal and stemness in AM- and WJ-MSCs under basal condition. (**A**) Stemness, (**B**) cell adhesion and (**C**) self-renewal genes. Data represented mean counts ± SD. Asterisks (*) and (**) represent statistical significance of *p* < 0.05 and *p* < 0.005, respectively.

Out of 199 selected genes including six housekeeping genes, 102 and 111 genes were detected in AM-MSCs and WJ-MSCs, respectively, under non-induced conditions. Nine genes were only detectable in WJ-MSCs. They are *APH1A*, *CTBP2*, *FZD8*, *GAS1*, *KAT2B*, *NCAM1*, *PRKCE*, *PSEN2* and *STK36* (Figure S1, see Supplementary Material). Both MSCs expressed stemness-related genes with no significant differences (Figure 1A). In comparison, adhesion genes (*CDH2* and *NCAM1*) were significantly higher in WJ-MSCs (Figure 1B), suggesting that they could adhere to the surface better than AM-MSCs. Whilst for self-renewal, most genes were comparably expressed in both MSCs except for *ADAR*, *CXCL12* and *PAFAH1B1* (Figure 1C).

**Figure 2:**
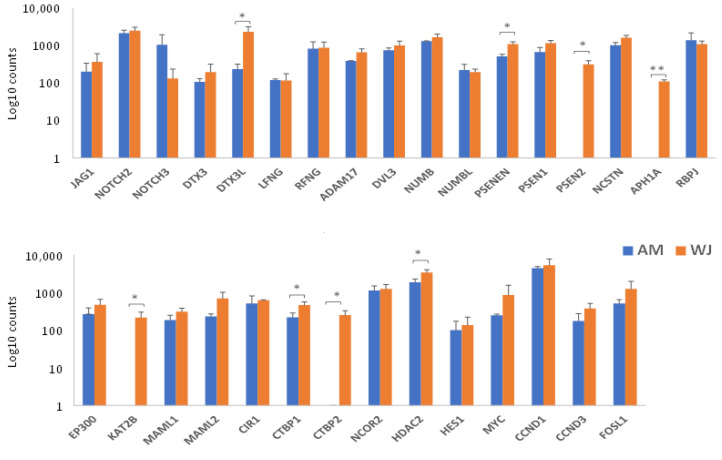
Notch signalling pathway-related gene expression in AM- and WJ-MSCs under basal condition. Data represented mean counts ± SD. Asterisks (*) and (**) represent statistical significance of *p* < 0.05 and *p* < 0.005, respectively.

**Figure 3:**
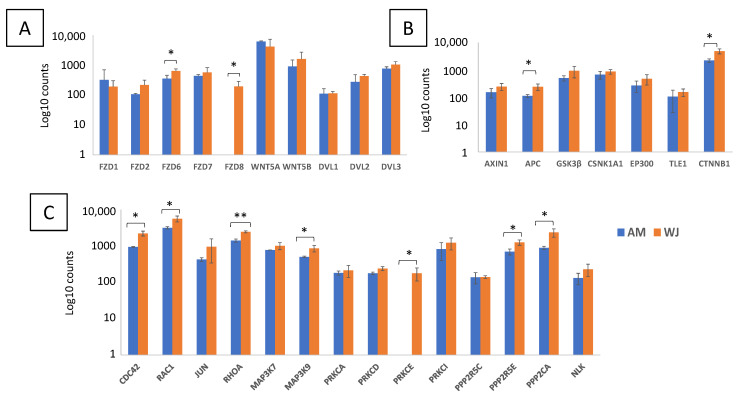
Wnt signalling pathway-related gene expression in AM- and WJ-MSCs under basal condition. (**A**) Genes encoding Wnt ligands and receptors, (**B**) genes encoding canonical Wnt pathway and (**C**) non-canonical Wnt pathway. Data represented mean counts ± SD. Asterisks (*) and (**) represent statistical significance of *p* < 0.05 and *p* < 0.005, respectively.

**Figure 4:**
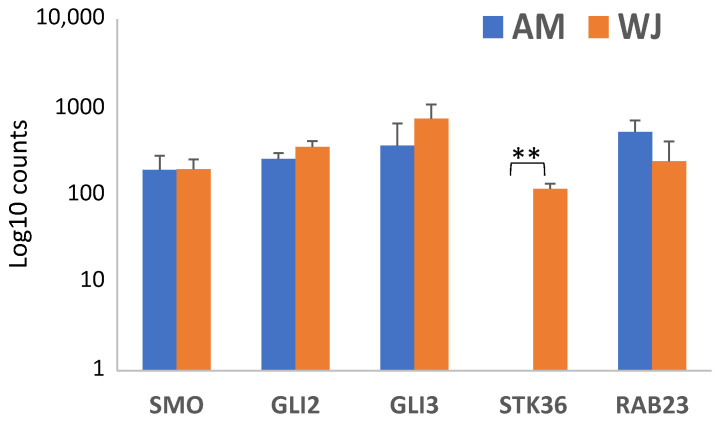
Hedgehog signalling genes expressed in both AM- and WJ-MSCs under basal condition. Data represented mean counts ± SD. Asterisks (**) represent statistical significance *p* < 0.005.

All 31 genes involved in the Notch signalling pathway were detected in WJ-MSCs. The expression of *PSEN2*, *APH1A*, *KAT2B* and *CTBP2* was undetectable in AM-MSCs (Figure 2). Other significant differentially expressed genes found in WJ-MSCs were *DTX3L*, *PSENEN*, *CTBP1*, and *HDAC2*. All genes associated with Wnt pathways were expressed in WJ-MSCs (Figure 3). The expression of *FZD8* and *PRKCE* was immeasurable (Figure 3A,C) but all genes involved in the canonical pathway were expressed in AM-MSCs (Figure 3B). Besides that, the significantly expressed genes were found in WJ-MSCs compared to AM-MSCs. For example, WJ-MSC had profound expression in genes involved in the Wnt-planar cell polarity pathway (*CDC42*, *RAC1*, *RHOA*, *MAPK9*) and Wnt-Ca^2+^ pathway (*PRKCE*, *PPP2R5E*, *PPP2CA*) (Figure 3C). Surprisingly, hedgehog ligands (*SHH*, *DHH*, *IHH*) were not expressed, while its transcription factors (*GLI 2*, *GLI3*) and other genes were equally expressed in both MSCs (Figure 4). These results indicated that WJ-MSCs could be more responsive to the activation of signalling pathways than AM-MSCs.

### 3.2. Differential Gene Expression of AM- and WJ-MSCs upon Osteogenic Induction

**Figure 5:**
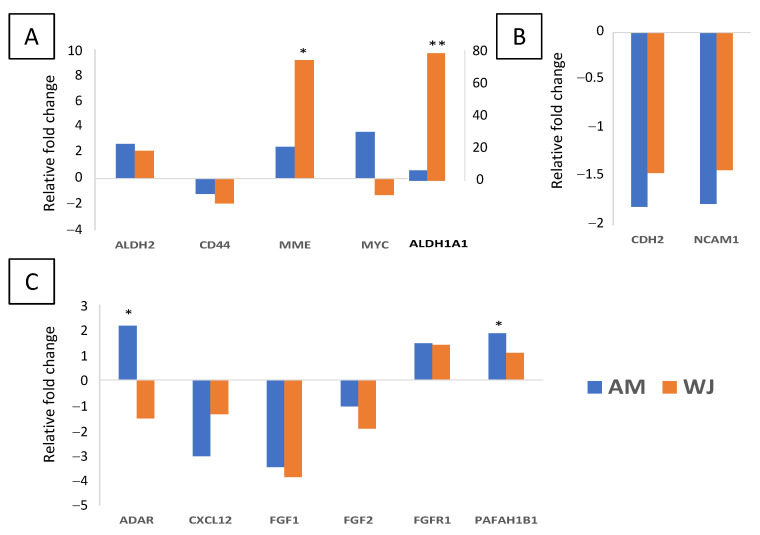
Differential expression of genes associated with self-renewal and stemness in AM- and WJ-MSCs during osteogenesis. (**A**) Stemness, (**B**) cell adhesion and (**C**) self-renewal genes. The y-axis on the right side in (**A**) indicates the expression level of *ALDH1A1* only. Asterisks (*) and (**) represent statistical significance of *p* < 0.05 and *p* < 0.005, respectively.

Next, we investigated the differential gene expression profiles of AM- and WJ-MSCs during osteogenesis through osteogenic induction. After induction, more genes were upregulated with >1.5-fold change in AM-MSCs than WJ-MSCs (Table S1). A decrease in *CD44* expression was observed in both MSCs, while the expression of *MYC* was inversely expressed (Figure 5A). Most stemness genes were upregulated, especially *ALDH1A1* and *MME*, which were significantly increased in WJ-MSCs during osteogenesis. In contrast, the expression of adhesion genes (*CDH2*, *NCAM1*) was reduced upon induction (Figure 5B). Osteogenesis also caused downregulation (*CXCL12*, *FGF1*/*2*) in both MSCs. *ADAR* and *PAFAH1B1* were significantly elevated in AM-MSCs. However, a decrease in *ADAR* was observed in WJ samples (Figure 5C).

**Figure 6:**
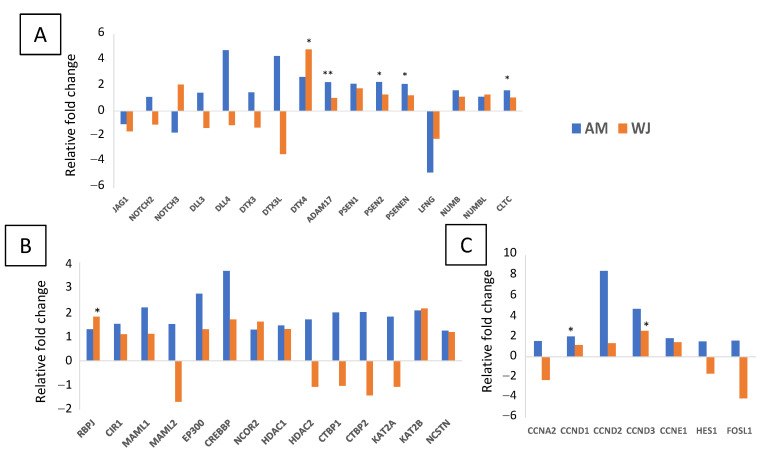
Differential expression of genes associated with Notch signalling pathways in AM- and WJ-MSCs during osteogenesis. (**A**) Ligands, receptors, ubiquitin E3 ligases and proteases; (**B**) other coregulators; and (**C**) downstream target genes. Asterisks (*) and (**) represent statistical significance of *p* < 0.05 and *p* < 0.005, respectively.

In comparison to WJ-MSCs, more genes associated with the Notch pathway were upregulated in AM-MSCs during osteogenesis (Figure 6). They shared the same expression profiles for *JAG1* and *DTX4* but a distinct expression for *DLL* ligands and other ubiquitin E3 ligases, *DTX*s (Figure 6A). It is clear that they had different preferences in receptor activation; AM-MSCs showed an increase in *NOTCH2*, while *NOTCH3* was upregulated in WJ-MSCs. There was downregulation of the lunatic fringe gene (*LFNG*) and significant increase in *ADAM17* expression in AM samples. Subunits of the γ-secretase complex, the *PSENEN* and *PSEN* expression was also higher in AM-MSCs. RBPJ acts as an activator when bound to Notch. Although its expression was higher in WJ-MSCs, chromatin remodelling complexes recruited by RBPJ, such as histone acetyltransferases (*KAT2A*) and histone deacetylase (*HDAC2*), were downregulated during osteogenesis compared to AM-MSCs (Figure 6B). Besides that, they had an opposite expression profile of *CTBP1/2* and *MAML2*. In addition, different subsets of Notch target genes were activated. For instance, all were increased in AM-MSCs; however, the expression of *HES1*, *FOSL1* and *CCNA2* was downregulated in WJ-MSCs after osteo-induction (Figure 6C). These results demonstrated that Notch activation during WJ-MSCs osteogenesis could be DLL/JAG-independent; it is unclear whether this led to downregulation of some target genes.

**Figure 7:**
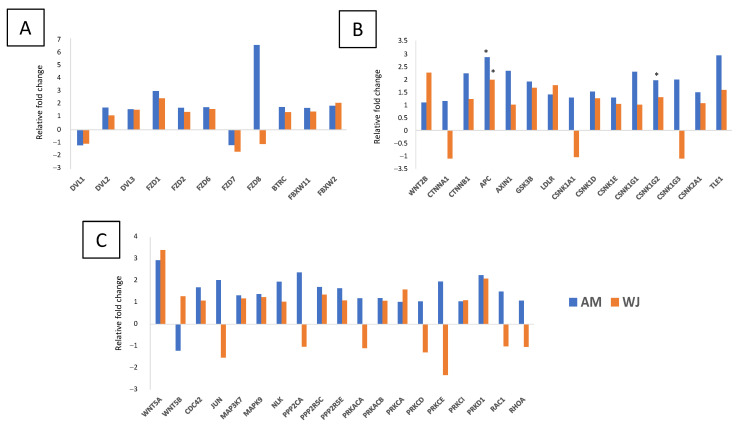
Differential expression of genes associated with Wnt signalling pathways in AM- and WJ-MSCs during osteogenesis. (**A**) Receptors, ubiquitin ligases, (**B**) canonical pathway and (**C**) non-canonical pathway. Asterisks (*) represent statistical significance *p* < 0.05.

More Wnt-associated genes have shown higher expression when AM-MSCs are undergoing osteogenesis compared to WJ-MSCs (Figure 7). Both MSCs had comparable gene expression patterns for *DVL*s, ubiquitin ligases and FZD receptors except *FZD8* (Figure 7A). However, their profound differences were noticeable in genes regulating canonical and non-canonical pathways where fewer genes were upregulated (>1.5-fold change) in WJ samples. Both MSCs expressed elevated *WNT2B* and *WNT5A* but *WNT5B* was decreased upon osteogenic induction in AM-MSCs (Figure 7B). However, only the expression of *β-catenin* was increased while *α-catenin* was downregulated in WJ samples. More inverted expression profiles were observed in the non-canonical Wnt pathway whereby upregulation of protein phosphatases (*PPP2CA*, *PRKCD/E*, *PRKACA*) and downstream target genes (*JUN*, *RAC1*) were found in AM-MSCs (Figure 7C). These results suggested that the non-canonical pathway was more profound for AM-MSCs during osteogenic differentiation. Canonical Wnt signalling could be more predominant in WJ-MSCs, judging by the downregulation of negative regulators of Wnt/β-catenin (*CTNNA1*, *CSNK1A1*) and *RAC1*/*JUN* involved in the non-canonical pathway.

**Figure 8:**
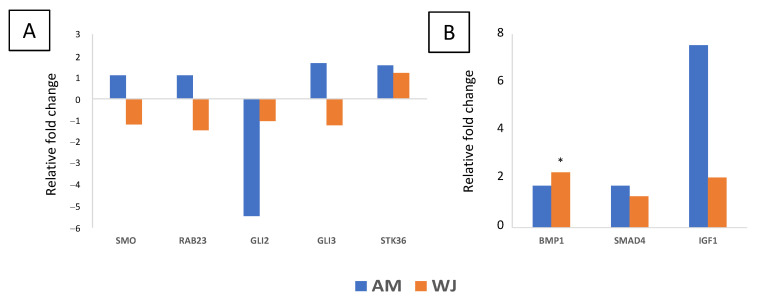
Differential expression of genes associated with (**A**) Hedgehog and (**B**) other signalling pathways in AM- and WJ-MSCs during osteogenesis. Asterisks (*) represent statistical significance *p* < 0.05.

All genes associated with Hh signalling were downregulated in WJ-MSCs except the positive regulator (*STK36*) of GLI transcription factor. On the other hand, AM-MSCs showed increases in positive regulators such as *SMO* and *STK36*, transcription factor *GLI3* as well as a negative regulator (*RAB23*) during osteogenesis (Figure 8A). Nevertheless, genes associated with other signalling pathways such as *BMP1*, *SMAD4* and *IGF1* were upregulated in both MSCs upon osteo-induction (Figure 8B). *IGF1* expression was initially undetectable in both MSCs at the undifferentiated condition. Thus, it is worth mentioning that IGF1/SMAD signalling might be important during osteogenesis, especially in AM-MSCs, as it has been shown to be essential in osteoblast differentiation in MSCs [20].

## 4. Discussion

Placental tissues have become one of the most utilized MSCs for tissue engineering and regenerative therapy. They are considered the best sources due to the lack of ethical concerns, non-invasive isolation, and ease of availability. Evidence has shown that the origin of MSCs is one of the determinant factors in their biological properties and therapeutic efficacies [21,22]. Biological discrepancies were also observed in MSCs derived from different compartments of the umbilical cord [23].

Our findings revealed that they not only possessed distinct gene expression profiles under undifferentiated status, but also committed to osteogenic lineage by activating discrete signalling molecules. For example, WJ-MSCs had a higher *PAFAH1B1* expression level than AM-MSCs. A previous study showed that PAFAH1B1, also known as Lissencephaly 1 protein (LIS1), enhanced cadherins accumulation in the stem cell niche that mediated cell adhesion and self-renewal [24]. This was accordantly supported by significantly higher expressions of cadherin and adhesion molecules (*CDH2* and *NCAM1*) in WJ-MSCs. CDH2 was expressed mostly in mesenchymal tissues and served as a regulator in determining stem cell fate via FGF signalling [25]. In addition, *ALDH1A1* and *NCAM1* have also been found to be tissue-specific genes in placental MSCs [26].

Most of the genes involved in the cascade of Wnt, Notch and Hh signal transduction were expressed in both MSCs with varying magnitude in some genes when they were unstimulated. Both MSCs expressed the most evaluated genes involved in Wnt signalling pathways at the basal condition. The expression of non-canonical Wnt signalling molecules such as *WNT5A* and *WNT5B* was more profound compared to those involved in the canonical pathway. Batsali et al. [13] demonstrated that genes involving the non-canonical Wnt pathway were upregulated while those associated with the canonical route were reduced in WJ-MSCs compared to BM-MSCs. Activation of Wnt signalling in UC-MSCs was found to promote proliferation and maintain self-renewal while restricting lineage differentiation [27]. In view of the expression from ligands (*NOTCH2*, *NOTCH3*), frizzled receptors and intracellular molecules, Notch signalling was concomitantly activated in both MSCs, although some genes (*PSEN2*, *APH1A*, *CTBP2*, *KAT2B*) were solely detected in WJ-MSCs. The expressions of *Notch1-3* and *Dll1* have been reported in liver-derived MSCs [28]. In contrast, Hh pathways seemed to be less important in undifferentiated status, judging by the undetectable expression of ligands such as *SHH*, *IHH* and *DHH* in both MSCs despite noticeable expression of downstream targets, such as *GLI2*, *GLI3*, *SMO* and *RAB23*. However, the involvement of non-canonical Hh signalling could not be excluded because SMO and PTCH1 receptors can activate GLI transcription factors directly or indirectly without the presence of Hh ligands [29]. Taken together, signalling pathways such as Wnt, Notch and BMP are required to maintain the characteristics of undifferentiated AM- and WJ-MSCs. Differential expression of Wnt- and Notch-associated genes leads us to speculate that WJ-MSCs may acquire superior commitment in lineage differentiation than AM-MSCs.

After investigating their intrinsic differences in basal condition, we further examined their transcriptomic responses to osteogenesis. Osteogenesis utilizes multiple signalling pathways to induce undifferentiated MSCs to osteoblast formation. From our observation, WJ-MSCs might be more responsive to extrinsic osteogenic factors, thus, the induction period was shortened compared to AM-MSCs (Figure S2). Upon induction, the upregulation of self-renewal genes was observed in AM-MSCs (*ADAR*, *PAFAH1B1*, *RB1*) as well as WJ-MSCs (*ALDH1A1*, *MME*). This is not unusual, as the dual functions of these genes have been reported previously. For instance, besides maintaining self-renewal and cell survival, ADAR knockdown has affected osteoblast differentiation in mice [30]. Transcriptomic analysis demonstrated that PAFAH1B1 was downregulated by osteogenic BMPs in MSCs [31]. Besides that, ALDH^+^ WJ-MSCs were found to express higher osteogenic genes such as *RUNX2*, *OSX* and *OPN* [32]. Nallamshetty et al. [33] demonstrated that during primary MSCs’ osteoblastogenesis, ALDH1A1 was also expressed besides bone marker alkaline phosphatase (ALP). MME, also known as CD10, was proposed to be a key biomarker for the human UC stromal stem cell [34]. Recently, CD10^+^ expressing perivascular stem cells exhibited higher proliferation and osteogenic potential [35].

**Figure 9:**
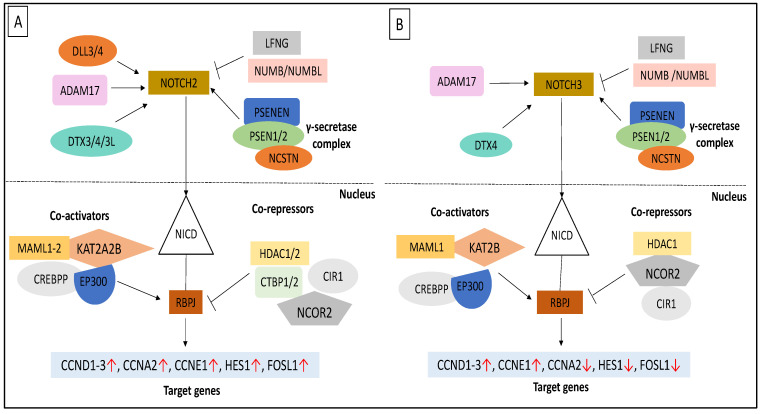
Notch signalling pathway in (**A**) AM-MSCs and (**B**) WJ-MSCs after osteogenic induction. Symbol ↑, ↓ and ׀˗˗˗ represent upregulation, downregulation and inhibition, respectively. NICD: Notch intracellular domain.

During osteogenesis, Notch activation in AM-MSCs is evident by the upregulation of many genes involved in the cascade from signalling molecules down to intracellular and target genes but the signal transduction occurred in WJ-MSCs has been streamlined. As shown in Figure 9A, osteogenic initiation was triggered by the binding of ligands *JAG2* to the *NOTCH4* receptor, enhancing the expression of γ-secretase complex (*PSENEN*, *PSEN1-2*), which further propagated the signal to co-activators such as *EP300*, *MAML1/2*, and *KAT2B*, leading to the increased expression of target genes (*HES1*, *CCND1-3*, *CCNE1*, *FOSL1*). Osteogenic promotion of Notch signalling in AM-MSCs could also be caused by the upregulation of *NOTCH4*. Conversely, the Notch signalling was initiated through the NOTCH3 receptor which was cleaved by γ-secretase and ubiquitinated by E3 ubiquitin ligase *DTX4* only in WJ-MSCs (Figure 9B). We postulated that non-canonical Notch signalling was stimulated in WJ-MSCs without the presence of DLL ligands. Nonetheless, this upstream signal was insufficient to activate a broad range of target genes like what occurred in AM-MSCs. Previous studies showed that Deltex acted as a positive regulator of Notch signalling. DTX1 regulated transcription independently of *RBPJ* and *HES1* through co-activator *EP300* [36]. Increased expression of Notch 1-2 and Dll1 was shown to be important in initiating osteogenic differentiation, while elevating Notch 3 expression occurred later in rat liver MSCs [28]. Controversial results indicated that Notch signalling has a dual function in osteoblastic differentiation whereby in vivo studies showed that the RBPjκ-dependent Notch signalling pathway suppressed osteogenesis [37]. These coordinated signalling events support faster osteogenic induction observed in WJ-MSCs than AM-MSCs, requiring a longer induction period.

**Figure 10:**
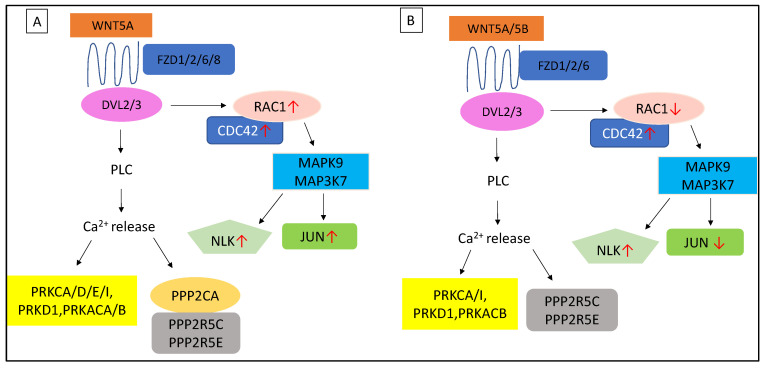
Non-canonical Wnt pathways in (**A**) AM-MSCs and (**B**) WJ-MSCs after osteogenic induction. Symbol ↑ and ↓ represent upregulation and downregulation, respectively. PLC: Phospholipase C.

Wnt signalling has always played a critical role in the development and maintenance of a large variety of tissues and organs, especially bone. In addition, Notch and Wnt signals could crosstalk and act synergistically to support cell proliferation and differentiation in osteoprogenitor cells [38]. Upon osteogenic induction, both MSCs utilized slightly different signal molecules to activate canonical and non-canonical Wnt pathways as depicted in Figure 10. Non-canonical Wnt pathways might have a more critical role in AM-MSCs during osteogenesis due to high expression of the subunits of the destruction complex (*CSNK1G1-3*, *CSNK1A1L*, *CSNK1D*, *APC*, *AXIN1*) which negatively regulate Wnt/β-catenin signalling by degrading β-catenin and preventing it from entering the nucleus [39]. More intracellular molecules associated with the Wnt-Ca^2+^ pathway was expressed such as *PPP2CA*, *MAP3K71P1* and *PRKCE*. In addition, upregulation of *RAC1* and *JUN*, which are involved in the Wnt-PCP pathway, was also noticeable in AM-MSCs. According to Lojk and Marc [40], canonical and non-canonical pathways share similar extracellular and membrane components despite their distinct intracellular molecules. Their activation is determined by the amount of Wnt ligands, receptors, coreceptors and inhibitors expressed during cell differentiation.

The hedgehog (Hh) pathway modulates bone formation and homeostasis. It is activated through the key signal transducer SMO and GLI transcription factors. RAB23, a suppressor of Hh signalling was found to inhibit GLI function through interaction with SUFU, another negative regulator of Hh signalling. In the absence of Hh ligands, GLI is converted to repressor form, thus preventing signal transduction [41,42]. However, the SHH non-canonical signalling cascade, which is GLI-independent, could occur via SMO-dependent or independent route [43]. Due to the downregulation of *GLI1-3* and *SMO* in WJ-MSCs, conventional Hh activation was unlikely, but osteogenesis via a non-canonical signalling route could not be excluded as the expression level of cyclins (*CCND1-3*, *CCNE1*) was noticeably increased. High expression of miR-342-3p in UC-MSCs increased the expression of osteogenic-related genes via activation of the SHH pathway [44]. In addition, positive regulation of Hh signalling in MSCs’ osteogenesis might produce conflicting effects on MSCs from other sources [14].

BMP1 and BMP2 have been shown to play a crucial role in inducing osteogenesis and matrix remodelling in BM-MSCs [45,46]. *BMP1* expression was only detected in both MSCs under basal and osteogenic conditions but not *BMP2* or *BMP3*, suggesting it has a predominant role in promoting osteogenesis in these prenatal MSCs. Compared to BMP2/6/7/9, the role of BMP1 in promoting osteoblastic differentiation of MSCs is still lacking. However, the ablation of BMPI caused osteogenesis imperfecta [47,48], while its overexpression promoted osteogenesis [46]. The expression of *SMAD4*, a mediator of the BMP/TGFβ pathway, was also upregulated. SMAD4 knockout in mice was shown to decrease bone mineralization during osteoblast stages [49]. In addition, SMAD could also induce osteogenesis by binding directly to a Taz protein via a TGFβ-independent manner [50].

In conclusion, our results demonstrated that some unique genes are only expressed in these MSCs in basal or osteogenic conditions. Substantial genes involved in Wnt, Notch and Hh signalling were expressed during osteogenesis in AM-MSCs. WJ-MSCs seemed to be more responsive upon osteo-induction, as most genes were found highly expressed when they were undifferentiated. This study has provided insights into the intrinsic biological differences that may regulate and orchestrate osteogenic cell fate in MSCs. Nevertheless, more investigation is required to decipher how these discrepancies contribute to their functionality in treating bone diseases.

## Data Availability

Data are available upon request.

## Supplementary Materials

The following supporting information can be download at: https://www.mdpi.com/article/10.3390/cimb44090291/s1, Figure S1: Genes detected in AM- and WJ-MSCs under basal condition; Figure S2: Osteogenic differentiation of the isolated AM- and WJ-MSCs. AM- and WJ-MSCs were stained with Alizarin Red. Scale bar = 200 µm; Table S1: Fold change of genes upregulated and downregulated upon osteo-induction in AM-and WJ-MSCs.
