# Supplementary material for: Gene Expression Profiles of Human Mesenchymal Stromal Cells Derived from Wharton’s Jelly and Amniotic Membrane before and after Osteo-Induction Using NanoString Platform
Source: Curr Issues Mol Biol. 2022 Sep 14;44(9):4240–54. doi: 10.3390/cimb44090291 (PMC9497674; doi:10.3390/cimb44090291)
Supplement: Supplementary file 1 [file cimb-44-00291-s001.zip › cimb-1835023-supplementary.pdf]

Gene expression profiles of human mesenchymal stromal cells derived from Wharton's jelly and amniotic membrane before and after osteo-induction using NanoString platform

Vun Vun Hiew, Haselamirrah Mohd Akhir, Peik Lin Teoh*

Biotechnology Research Institute, Universiti Malaysia Sabah, Jalan UMS, 88400 Kota Kinabalu, Sabah, Malaysia

*Corresponding author: peiklin@ums.edu.my

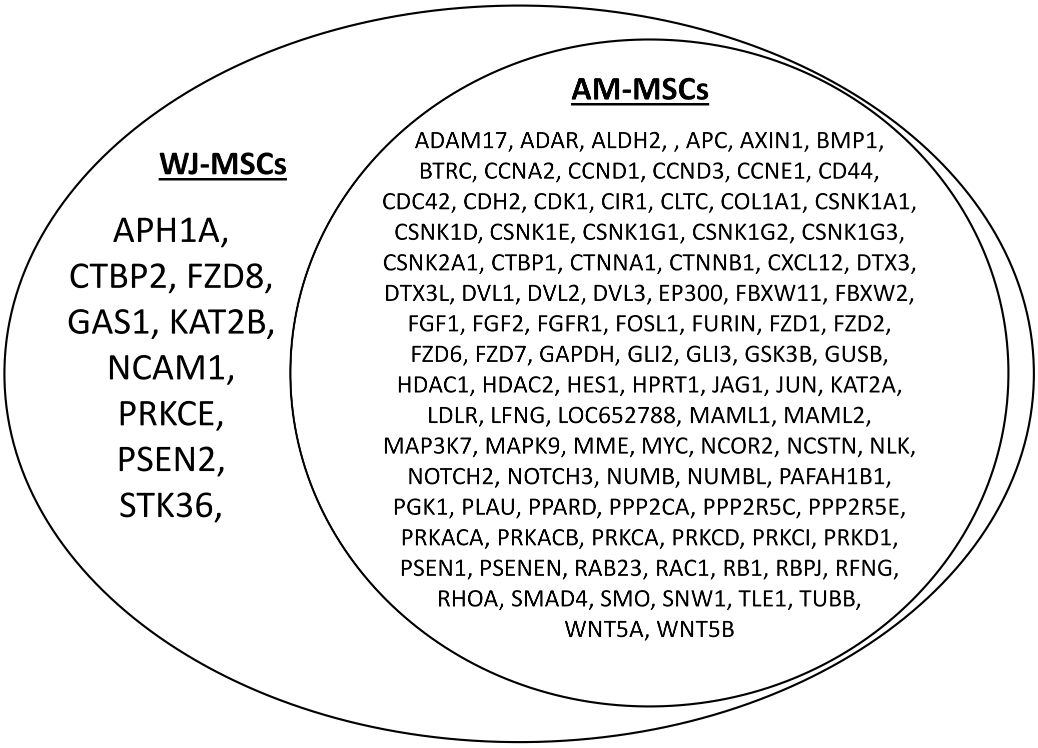

Figure S1. Genes detected in AM- and WJ-MSCs under basal condition.

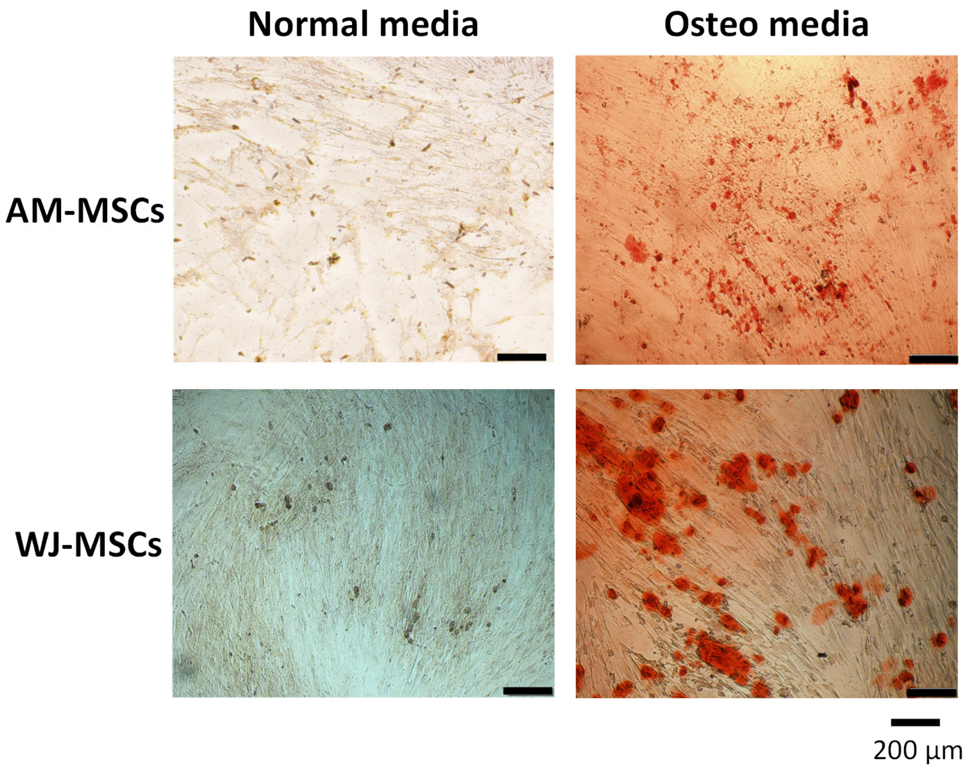

Figure S2. Osteogenic differentiation of the isolated AM- and WJ-MSCs. AM- and WJ-MSCs were stained with Alizarin Red. Scale bar = 200 µm.

Table S1. Fold change of genes upregulated and downregulated upon osteo-induction in AM- and WJ-MSCs.

**AM-MSCs**						**WJ-MSCs**					
**Upregulated**				**Downregulated**		**Upregulated**				**Downregulated**	
ADAM17	2.25	HES1	1.52	CD44	-1.19	ADAM17	1.03	PGK1	1.57	ADAR	-1.53
ADAR	2.15	HPRT1	1.08	CDH2	-1.81	ALDH1A1	78.28	PPARG	2.30	CCNA2	-2.29
ALDH1A1	6.50	IGF1	7.55	CDK1	-1.32	ALDH2	2.14	PPP2R5C	1.35	CD44	-1.92
ALDH2	2.68	JUN	2.02	COL1A1	-1.24	APC	1.97	PPP2R5E	1.09	CDH2	-1.46
APC	2.85	KAT2A	1.79	CXCL12	-3.04	AXIN1	1.00	PRKACB	1.07	CDK1	-2.85
AXIN1	2.32	KAT2B	2.05	DVL1	-1.18	BMP1	2.29	PRKCA	1.59	COL1A1	-1.10
BMP1	1.74	LDLR	1.40	FGF1	-3.47	BTRC	1.35	PRKCI	1.10	CSNK1A1	-1.04
BTRC	1.73	LOC652788	1.04	FGF2	-1.06	CCND1	1.16	PRKD1	2.09	CSNK1G3	-1.10
CCNA2	1.54	MAML1	2.17	FZD7	-1.17	CCND2	1.35	PSEN1	1.77	CTBP1	-1.02
CCND1	2.00	MAML2	1.49	GUSB	-1.02	CCND3	2.56	PSEN2	1.29	CTBP2	-1.41
CCND2	8.47	MAP3K7	1.32	ISL1	-1.15	CCNE1	1.42	PSENEN	1.22	CTNNA1	-1.10
CCND3	4.73	MAPK9	1.38	JAG1	-1.01	CDC42	1.08	RB1	1.30	CXCL12	-1.36
CCNE1	1.84	MME	2.46	KRT15	-7.41	CIR1	1.08	RBPJ	1.79	DLL3	-1.31
CDC42	1.69	MYC	3.60	LFNG	-4.78	CREBBP	1.68	RFNG	1.26	DLL4	-1.09
CIR1	1.50	NCOR2	1.26	NCAM1	-1.78	CSNK1D	1.25	SMAD4	1.30	DTX3	-1.28
CREBBP	3.66	NCSTN	1.22	NOTCH3	-1.66	CSNK1E	1.03	STK36	1.20	DTX3L	-3.35
CSNK1A1	1.28	NLK	1.95	RAB23	-5.46	CSNK1G1	1.00	TLE1	1.58	DVL1	-1.07
CSNK1D	1.51	NOTCH2	1.09	RFNG	-1.17	CSNK1G2	1.29	WNT2B	2.25	FGF1	-3.87
CSNK1E	1.28	NUMB	1.62	WNT5B	-1.22	CSNK2A1	1.06	WNT5A	3.40	FGF2	-1.94
CSNK1G1	2.29	NUMBL	1.12			CTNNB1	1.22	WNT5B	1.28	FOSL1	-4.10
CSNK1G2	1.95	PAFAH1B1	1.85			DTX4	4.80			FZD7	-1.67
CSNK1G3	1.98	PGK1	1.03			DVL2	1.10			FZD8	-1.09
CSNK2A1	1.48	PLAU	1.48			DVL3	1.53			GLI2	-1.04
CTBP1	1.97	PPARD	1.42			EP300	1.28			GLI3	-1.23
CTBP2	1.98	PPARG	4.12			FBXW11	1.39			HDAC2	-1.06
CTNNA1	1.14	PPP2CA	2.37			FBXW2	2.05			HES1	-1.66
CTNNB1	2.22	PPP2R5C	1.71			FGFR1	1.40			HPRT1	-1.58
DLL3	1.42	PPP2R5E	1.65			FURIN	1.44			ISL1	-1.23
DLL4	4.74	PRKACA	1.19			FZD1	2.40			JAG1	-1.58
DTX3	1.45	PRKACB	1.20			FZD2	1.36			JUN	-1.54
DTX3L	4.29	PRKCA	1.02			FZD6	1.59			KAT2A	-1.06
DTX4	2.66	PRKCD	1.05			GAS1	6.56			KRT15	-1.47
DVL2	1.69	PRKCE	1.96			GSK3B	1.66			LFNG	-2.15
DVL3	1.57	PRKCI	1.05			HDAC1	1.29			MYC	-1.27
EP300	2.73	PRKD1	2.25			IGF1	2.08			NCAM1	-1.43
FBXW11	1.67	PSEN1	2.13			KAT2B	2.13			NOTCH2	-1.05
FBXW2	1.83	PSEN2	2.27			LDLR	1.76			PLAU	-13.4
FGFR1	1.45	PSENEN	2.11			LOC652788	1.03			PPARD	-1.29
FOSL1	1.58	RAC1	1.50			MAML1	1.09			PPP2CA	-1.03
FURIN	1.17	RB1	1.79			MAML2	1.67			PRKACA	-1.10
FZD1	2.96	RBPJ	1.28			MAP3K7	1.18			PRKCD	-1.29
FZD2	1.68	RHOA	1.08			MAPK9	1.24			PRKCE	-2.34
FZD6	1.72	SMAD4	1.74			MME	9.14			RAB23	-1.46
FZD8	6.48	SMO	1.09			NCOR2	1.59			RAC1	-1.02
GAS1	16.34	SNW1	2.00			NCSTN	1.17			RHOA	-1.04
GLI2	1.65	STK36	1.34			NLK	1.03			SMO	-1.20
GLI3	1.55	TLE1	2.92			NOTCH3	2.06			SNW1	-1.15
GSK3B	1.90	WNT2B	1.09			NUMB	1.11				
HDAC1	1.44	WNT5A	2.94			NUMBL	1.29				
HDAC2	1.68					PAFAH1B1	1.08				
